# The Diagnostic and Therapeutic Dilemma of Seronegative Pulmonary Renal Syndrome: A Case Report

**DOI:** 10.7759/cureus.40634

**Published:** 2023-06-19

**Authors:** Abubakr O Bajaber, Abdullah S Binsaeedu, Aiman G Muqrad, Ahmed Elsharkawy, Ahmad Alghitany

**Affiliations:** 1 College of Medicine, Alfaisal University, Riyadh, SAU; 2 Department of Medicine, Saudi German Hospital, Riyadh, SAU; 3 Department of Nephrology, Ain Shams University, Cairo, EGY

**Keywords:** case report, pediatric, idiopathic, seronegative, membranous nephropathy, diffuse alveolar hemorrhage, pulmonary renal syndrome

## Abstract

Pulmonary renal syndrome (PRS) is a combination of rapid progressive glomerulonephritis (RPGN) and diffuse alveolar hemorrhage (DAH) caused by a variety of immunological and non-immunological etiologies. The difficulty in identifying and reporting seronegative PRS cases could be attributed to the lack of specific immunological markers. Thus, we report a rare case of a 13-year-old boy who was initially diagnosed with idiopathic pauci-immune pulmonary capillaritis (IPIPC). A year later, his condition became complicated, and was referred for further workup. During his hospital stay, he underwent a renal biopsy that showed stage II membranous nephropathy (MN). He tested negative for immunological markers and a diagnosis of seronegative PRS was established. He responded well to the immunosuppression therapy with monthly follow-ups. As in our patient, PRS may manifest as acute renal failure symptoms and non-specific respiratory symptoms that require extensive workup. The severity of the disease is inferred from the renal function at the time of presentation. Management involves immunosuppression and treatment of the underlying condition, with dialysis dependency occurring in a significant percentage of patients and a high mortality rate, especially in critically ill and older patients. In conclusion, timely diagnosis and treatment are essential given the condition’s rapid progression and high mortality rate.

## Introduction

Pulmonary renal syndrome (PRS) is defined as the combined clinical picture of rapid progressive glomerulonephritis (RPGN) and diffuse alveolar hemorrhage (DAH). It is, mostly, caused by a plethora of immunological etiologies and, to a lesser extent, by non-immunological etiologies [1]. Such a variety of potential causes might impose difficulty in identifying the exact etiology in PRS patients. Moreover, those diagnosed with a seronegative PRS are even more challenging to be identified and provided with targeted therapy as the potential etiology is idiopathic [1]. The proper diagnostic workup and management plan for seronegative patients are not established yet due to the scarcity of reported cases. Therefore, we present a rare pediatric case of seronegative PRS diagnosed in association with membranous nephropathy (MN).

## Case presentation

A healthy 13-year-old boy presented to the hospital emergency department with progressive shortness of breath, dry cough, and hemoptysis that had been ongoing for three weeks. The patient had no coronavirus disease 2019 (COVID-19) infection, and no vaccination was received. Upon admission, he was stable but exhibited slightly diminished air entry and skin pallor. Labs showed low hemoglobin (6.5 g/dL), elevated c-reactive protein (1.37 mg/dL) and erythrocyte sedimentation rate (49 mm/h), and normal leukocyte counts. Urinalysis was unremarkable and the renal profile (serum creatinine: 0.60 mg/dL, blood urea nitrogen: 22.85 mg/dL) was within normal ranges. Imaging revealed bilateral findings suggestive of pneumonia, while both bacterial and viral tests returned negative. The patient was admitted for a blood transfusion and antibiotics for symptomatic anemia and pneumonia. However, he experienced recurrent anemic episodes due to persistent hemoptysis and required multiple hospitalizations for blood transfusions. A diagnosis of idiopathic pauci-immune pulmonary capillaritis (IPIPC) was made based on the transbronchial biopsy findings. He was maintained on prednisolone (60 mg) and azathioprine (50 mg) for which he responded well, despite normal immunology panels.

**Figure 1:**
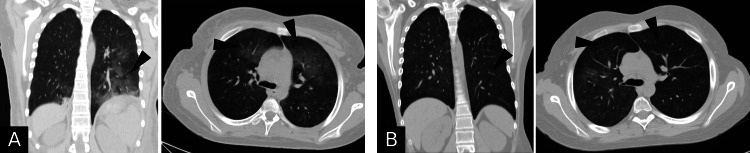
Chest computed tomography (CT) scan findings (coronal and transverse views; lung window) A: Baseline study upon hospitalization. Bilaterally scattered variable size patchy ground glass opacities (black indicators) showing central and peripheral distribution associated with crazy-paving pattern of interlobular septal thickening as well as peripheral alveolar consolidations. B: Follow-up study after discharge. Diffusely scattered bilateral fainting ground glass alveolar opacities (black indicators) indicating interval resolution of pulmonary findings.

A year later, the patient returned complaining of acute intense dyspnea. Imaging showed a pulmonary embolism and was started on enoxaparin sodium (80 mg). Later, he was referred to our hospital for further management. Upon admission, he was afebrile and vitally stable. He had a puffy edematous face and mild lower limb edema, and his chest computed tomography (CT) findings were suggestive of non-cardiogenic pulmonary edema (Figure 1A). His complete blood count showed mild anemia (hemoglobin: 9.9), coagulation profile was normal, and D-dimer (7 microgram/mL) was elevated. Urinalysis showed microscopic hematuria, mild proteinuria, and pus cells. Serum albumin level was low (1.33 g/dL), while total cholesterol and low-density lipoprotein (LDL) levels were (289 mg/dL) and (182 mg/dL), respectively (Table 1). Complements were low (C3: 57.5 mg/dL) and (C4: 12.6 mg/dL). Coombs tests were negative, and immunological panel sets were unremarkable (Table 2).

**Table 1:** Biochemical tests during hospitalization and follow-up Although there was a  mild deterioration during hospitalization, the patient’s biochemical profile improved toward discharge and normalized at the shown most recent follow-up visit. CRP, c-reactive protein.

	In-hospital						Follow-up
Days/Visit	1st	7th	15th	21st	27th	30th	­­—
Hemoglobin (g/dL)	9.9	7.6	7.8	8.3	10.4	9	12.5
Leukocytes (10^9^)	10.4	6.7	7.8	4.5	9.3	4.7	6.6
Albumin (g/dL)	1.33	2.15	3.67	2.39	2.7	2.64	4.10
Creatinine (mg/dL)	0.43	0.51	0.61	0.57	0.40	0.40	0.65
24 protein (mg/24 h)	700	—	—	—	—	37835	—
Diuresis (mL)	1400	—	—	—	—	2150	—
CRP (mg/L)	1.2	3.1	2.0	283.9	6.8	—	—

**Table 2:** Serological panels Serological test showed non-reactivity for the panels sent. ANA, antinuclear antibodies; p-ANCA, perinuclear anti-neutrophil cytoplasmic antibodies; MPO, myeloperoxidase; c-ANCA, cytoplasmic anti-neutrophil cytoplasmic antibodies; PR-3, proteinase-3; anti-dsDNA, anti-double stranded DNAA antibodies; LA1, lupus anticoagulant; anti-SSA, anti-Sjögren's-syndrome-related antigen A; anti-SSB, anti-Sjögren's-syndrome-related antigen B; IgM, immunoglobulin M; IgG,  immunoglobulin G; anti-GBM, anti-glomerular basement membrane; anti-PLA2R, anti-phospholipase A2 receptor; anti-THSD7A, anti-thrombospondin type I domain-containing 7A; anti-U1-snRNP, U1 small nuclear ribonucleoprotein particle antibodies.

Serology tests	1st panel	2nd panel	Reference ranges
ANA	<1:80	<1:80	<1.80 [Titer]
p-ANCA (MPO)	<1:2	—	<1:2 [Titer]
c-ANCA (PR-3)	<1:2	—	<1:2 [Titer]
Anti-dsDNA	<10	<10	<100 IU/mL
LA1	35.5	—	≤44 S
Anti-SSA/Ro	<1	<1	<7 U/mL
Anti-SSB/La	<1	<1	<7 U/mL
Anti-cardiolipin (IgM)	<2	<2	<12 U/mL
Anti-cardiolipin (IgG)	<2	<2	<12 U/mL
Anti-GBM	Tubular: <1:10; Glomerular: <2	—	Tubular: <1:10 [Titer]; Glomerular: ≤7 U/mL
Anti-PLA2R	<1:10	—	<1:10 [Titer]
Anti-THSD7A	<1:10	—	<1:10 [Titer]
Anti-U1-snRNP	<1	—	<5 U/mL
Anti-topoisomerase I (anti-Scl70)	<1	—	<7 U/mL
Anti-histidyl-tRNA synthetase (anti-Jo1)	<1	—	<7 U/mL
Light chains Kappa	14.6	—	3.3-19.4 mg/L
Light chains Lambda	9.2	—	5.7-26.3 mg/L

**Figure 2:**
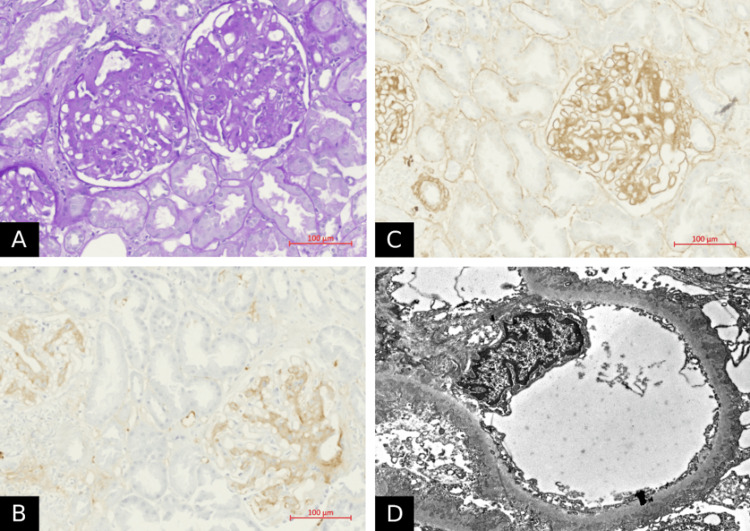
Renal biopsy findings show membranous nephropathy changes A, Periodic acid-Schiff stain; original magnification 40x. Multiple glomeruli with thickened basement membranes, mesangial matrix expansion, and mild hypercellularity. Few infiltrating inflammatory cells with minimal tubular atrophy and interstitial fibrosis. B, IgM immunohistochemistry; original magnification 40x. Presence of minimal IgM deposits in the mesangium only with no significant IgA deposits. C, IgG immunohistochemistry; original magnification 40x. A homogeneous strong granular presence of IgG in the mesangium. D, Electron micrograph; original magnification, 1000x. Strong osmiophilic deposits in the expanded mesangial matrix and on the outer subepithelial surface of the glomerular basement membrane due to matrix deposition. No organized deposits were identified. No subendothelial deposits were identified.

During his five-week hospital stay, the patient received two packed red blood cell transfusions and underwent a renal biopsy. The biopsy results showed stage II MN (Figure 2) but tested MN antibodies were negative. The patient was switched to apixaban (5 mg) for long-term pulmonary embolism prophylaxis. The patient was put off azathioprine and received multiple pulse steroid doses and two doses of cyclophosphamide (500 mg) as induction therapy. He was maintained on cyclosporine (100 mg). During hospitalization, he had one hemoptysis attack that resolved over three days but then became progressively febrile and was diagnosed with cytomegalovirus (CMV) infection and pneumonia on top of severe DAH. He needed supplemental oxygen but did not require intubation. After receiving broad-spectrum antibiotics and antiviral therapy, the patient's condition stabilized, and he remained on the prescribed course of antibiotics and antivirals according to the guidelines.

On discharge, the patient was hemoptysis-free and had normal renal function. He was discharged on prednisolone (50 mg) and cyclosporine (100 mg) and followed up for seven months without re-admission, except for an anemic episode (Hb 7 g/dL) due to a hemoptysis attack. Chest imaging showed improvement in pulmonary findings compared to that during hospitalization (Figure 1B). During follow-up, cyclosporine was up-titrated (125 mg) to achieve the desired trough blood level, and prednisolone was tapered down. The patient responded well with favorable clinical and biochemical parameters (Table 1). He is currently satisfied and back to his usual daily activities. He is planned to be kept on cyclosporine for two years with monthly follow-ups.

## Discussion

Although there is limited information on the epidemiology of PRS, the epidemiological distribution of the condition could be inferred from the causative etiologies. Anti-neutrophil cytoplasmic antibodies (ANCA)-associated vasculitis (AAV) and anti-glomerular basement membrane (anti-GBM) are the most common etiologies, constituting 60-70% and 10-20% of cases. Other immunological and non-immunological causes were also linked to PRS [1]. Recently, COVID-19 infection and vaccination have also been implicated in some cases, which could be attributed to the viral affinity for angiotensin-converting enzyme (ACE) receptors present in the pulmonary and renal tissues [1,2]. As our patient tested negative for immunological markers and had unremarkable history and investigation findings, a diagnosis of seronegative PRS was established. The lack of specific immunological markers leads to the underdiagnosis of such patients, which may contribute to the scarcity of reported cases and the delayed initiation of disease-specific treatment.

The severity of the disease is generally inferred from the renal function (i.e. creatinine level and dialysis dependence) at the time of presentation. Early diagnosis of PRS is crucial to avoid irreversible loss of renal function and severe pulmonary hemorrhage. It is important to exclude PRS-mimicking conditions and treat them accordingly [3]. Pulmonary and renal involvement should not necessarily occur simultaneously. Other systems' involvement depends on the underlying etiology [1].

In PRS, the kidneys are typically impacted to a greater extent, resulting in acute renal failure symptoms such as oliguria, hypertension, and edema. Urine dipstick and microscopy can reveal glomerulonephritis features, and the urine protein:creatinine ratio most often tends to be in the non-nephrotic range. Renal biopsy is the gold standard test, not only to confirm the diagnosis but to identify the nature of the nephropathy [1,4].

MN affects children at variable incidence rates (1.5%-8%) [5,6]. As in our case, MN serological antigens can be negative in 40% of cases, necessitating histopathological diagnosis, while novel antigens have been identified for some of the unidentified cases [7]. MN is linked with pulmonary embolism, more commonly than other nephrotic syndrome etiologies, which might explain our patient's presentation [8]. Conservative management is typically recommended for MN, with immunosuppression reserved for those with persistent high-grade proteinuria or a high risk of end-stage renal disease [9,10]. Despite being a common pathology, the association between MN and PRS remains unknown due to a lack of substantial evidence, highlighting the need for further investigation. Although cyclosporine is an effective treatment option for MN, rituximab has been proven to achieve better long-term remission; however, it was not utilized in our case due to unavailability [11]. Furthermore, MN prognosis is unpredictable with around 20-30% achieving spontaneous long-term remission, and our patient progressed well most likely due to the initiation of immunosuppressive therapy [10].

Pulmonary involvement in PRS patients is non-specific and may present as infection-like symptoms [1]. High-resolution CT is more reliable than chest X-ray for DAH detection [3]. Fiber-optic bronchoscopy and bronchoalveolar lavage are useful for diagnosing DAH, while lung biopsy is the gold standard but has high complication rates for which it is indicated only when a renal biopsy is precluded [1,3]. Severe respiratory symptoms may lead to respiratory failure, and mechanical ventilation is needed in about half of DAH patients [1].

Management of PRS depends on the severity of the disease and the underlying conditions. Nonetheless, immunosuppression is the mainstay of PRS management to achieve and maintain remission. Immunosuppression is, mainly, achieved using steroids and cyclophosphamide [12]. As the anti-GBM disease was the first reported etiology for PRS, the practice of providing plasma exchange for PRS patients was established. While plasma exchange has proven effective in treating anti-GBM patients, its benefits for AAV patients are limited, except in cases of severe renal dysfunction where it may contribute to improved renal recovery [1,13,14]. Further investigation is needed to determine its potential benefits for other PRS subgroups. Moreover, treatment of some causes may require alternative or additional measures, which highlights the need to identify the culprit when diagnosing PRS patients [1]. Furthermore, PRS patients with severe renal impairment are indicated for initiation of urgent hemodialysis, similarly, as for other kidney failure [1,4]. As in our patient, immunosuppressive therapy often leads to bacterial and CMV infections, which increase morbidity and mortality risk. Hence, careful monitoring and prompt initiation of antibacterial and antiviral therapy are crucial [15,16].

PRS has a high mortality rate (30-40%), especially in critically ill patients. A majority (>85%) of deaths occur during the acute phase, with older patients showing poorer outcomes [15]. Dialysis dependency occurs in 70-80% of patients within 1-2 years of diagnosis [1]. Prognosis varies based on age, baseline health status, severity, onset of treatment, and underlying etiology [1,15].

## Conclusions

In conclusion, PRS is a rapidly progressing and heterogeneous condition that typically presents with DAH and RPGN. Diagnosis requires an extensive workup to identify the underlying condition, and management varies based on the identified disease, with immunosuppression being the backbone. The nature of the linkage between MN and PRS, if exists, remains unknown due to limited literature, necessitating further investigation as more evidence accumulates. Idiopathic PRS patients are more challenging to manage, and plasma exchange is only established for the anti-GBM disease subtype. Immunosuppression-induced infection is a critical issue. Given the condition's heterogeneity, rapid progression, and high mortality rate, further research is necessary to establish standardized diagnostic and treatment protocols that can help reduce delayed diagnosis and ensure successful treatment, leading to improved prognosis.
